# A single session of moderate intensity exercise influences memory, endocannabinoids and brain derived neurotrophic factor levels in men

**DOI:** 10.1038/s41598-021-93813-5

**Published:** 2021-07-13

**Authors:** Blanca Marin Bosch, Aurélien Bringard, Maria G. Logrieco, Estelle Lauer, Nathalie Imobersteg, Aurélien Thomas, Guido Ferretti, Sophie Schwartz, Kinga Igloi

**Affiliations:** 1 Department of Neuroscience, Faculty of Medicine, University of Geneva, Geneva, Switzerland; 2 Department of Anesthesiology, Pharmacology and Intensive Care, Geneva University Hospitals, 1205 Geneva, Switzerland; 3 Unit of Toxicology, CURML, Lausanne University Hospital and Geneva University Hospitals, Geneva, Switzerland; 4 Faculty of Biology and Medicine, University of Lausanne, Chemin Vulliette 4, 1000 Lausanne, Switzerland; 5 Swiss Center for Affective Sciences, University of Geneva, Geneva, Switzerland; 6 Geneva Neuroscience Center, University of Geneva, Geneva, Switzerland; 7 Pulmonology Division, Geneva University Hospital, Geneva, Switzerland

**Keywords:** Hippocampus, Learning and memory, Long-term memory

## Abstract

Regular physical exercise enhances memory functions, synaptic plasticity in the hippocampus, and brain derived neurotrophic factor (BDNF) levels. Likewise, short periods of exercise, or acute exercise, benefit hippocampal plasticity in rodents, via increased endocannabinoids (especially anandamide, AEA) and BDNF release. Yet, it remains unknown whether acute exercise has similar effects on BDNF and AEA levels in humans, with parallel influences on memory performance. Here we combined blood biomarkers, behavioral, and fMRI measurements to assess the impact of a single session of physical exercise on associative memory and underlying neurophysiological mechanisms in healthy male volunteers. For each participant, memory was tested after three conditions: rest, moderate or high intensity exercise. A long-term memory retest took place 3 months later. At both test and retest, memory performance after moderate intensity exercise was increased compared to rest. Memory after moderate intensity exercise correlated with exercise-induced increases in both AEA and BNDF levels: while AEA was associated with hippocampal activity during memory recall, BDNF enhanced hippocampal memory representations and long-term performance. These findings demonstrate that acute moderate intensity exercise benefits consolidation of hippocampal memory representations, and that endocannabinoids and BNDF signaling may contribute to the synergic modulation of underlying neural plasticity mechanisms.

## Introduction

Regular physical exercise is a lifestyle factor, known to benefit neurocognitive functions and brain plasticity^1^, and which may reduce the risk of cognitive decline associated with Alzheimer’s disease^2^. Animal studies support that regular exercise fosters neurogenesis in the adult hippocampus and improves learning and memory^3^. In adult humans, neurogenesis in the hippocampus has been repeatedly suggested^4,5^, albeit being recently questioned^6^. Several lines of evidence converge to suggest that hippocampal synaptic plasticity is modulated, at least partially, by brain derived neurotrophic factor (BDNF)^7^. Specifically, physical exercise increases the levels of BDNF messenger RNA and protein in the hippocampus and other brain regions^7^, while blocking BDNF action in the hippocampus hinders the beneficial effect of exercise on memory^8^. Yet, the kinetics of exercise-related BDNF level changes are predominantly fast and transient^9^. In particular, BDNF levels rapidly increase in hippocampal subfields in response to exercise^10^, together with enhanced long-term potentiation (LTP) and synaptic plasticity^11^. These effects may mediate memory enhancement on the timescale of a few hours^12^.

In addition to raising BDNF levels, physical exercise yields a rapid increase in circulating endocannabinoids, which act on cannabinoid receptors CB1 and CB2^13–15^. Work in animal models have implicated endocannabinoid signaling in exercise-induced adult hippocampal neurogenesis^16^ and plasticity mechanisms^17^. Endocannabinoids directly mediate forms of retrograde plasticity^18^, and can also modulate other forms of plasticity including LTP^19^ and BDNF signaling^20^. One recent study directly linked endocannabinoid levels to memory enhancement and hippocampus function in mice by showing that blocking CB1 receptor in the hippocampus disrupted spatial memory performance, whereas artificially elevating endocannabinoid concentrations in sedentary animals increased BDNF levels and memory^21^.

In humans, short periods of exercise, or acute exercise, were reported to have diverse effects on memory and cognition, ranging from positive to detrimental^1,22–25^. These seemingly disparate findings may primarily be attributable to the use of different exercising intensities together with rather poor quantification of exercise intensities^26^ and between-subjects comparisons. Some of these exercising manipulations were done post-encoding as here^22,24^, while others performed the cognitive tasks after the exercise intervention^27,28^. A previous behavioral study showed that one 30-min session of moderate intensity exercise performed between the encoding and test session boosted associative memory consolidation^24^.

The main aims of the present study were (1) to confirm these effects using an individually-defined calibration for moderate (and high) physical effort, derived from a maximal effort test (VO_2max_), and (2) to unravel the underlying blood biomarker and neuroimaging correlates. Specifically, based on previous findings^24^, we hypothesized that moderate intensity exercise would yield memory benefits as compared to rest. We also asked whether such modulation of memory performance would be associated with exercise-related changes in BDNF and AEA levels. Based on the data reviewed above, we hypothesized that endocannabinoids and BDNF influence hippocampal functioning after acute physical exercise in humans, which we assessed using fMRI. Different exercising intensities have been used in previous work, but rarely compared and often poorly characterized. Grounded on previous results^24^, we hypothesized that moderate intensity exercise (corresponding here to cycling during 30 min at 65% of the maximal cardiac frequency measured during VO_2max_) would yield the largest benefits, especially at immediate test. Further, we expected that such memory benefits would be associated with exercise-related changes in BDNF and AEA levels. We also included an exploratory high intensity exercising condition corresponding to 15 min of exercise at 75% if the maximal cardiac frequency.

**Figure 1:**
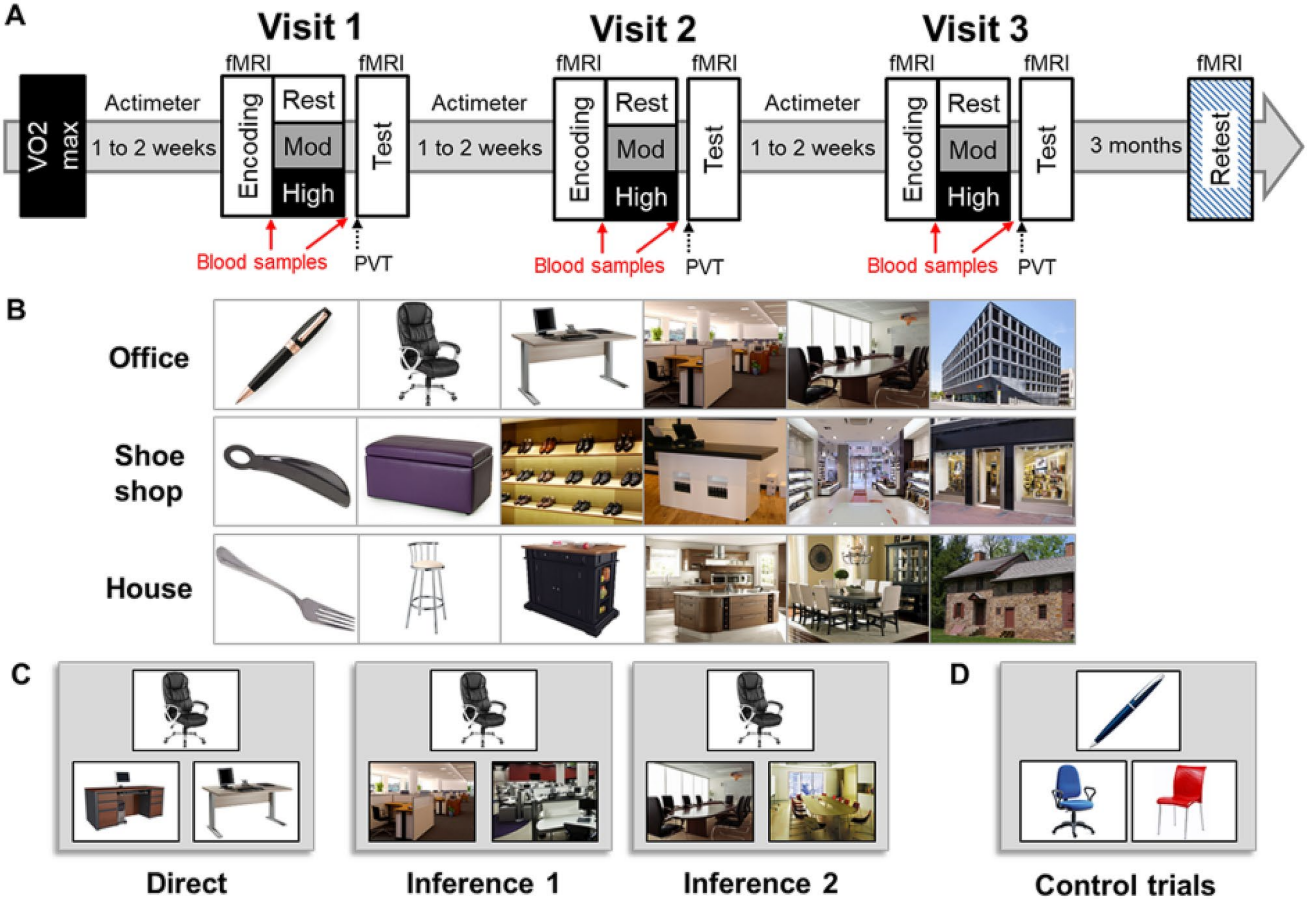
Experimental design and associative memory task. (**A**) Overview of the experimental protocol composed of a VO2max visit, followed by three experimental visits, and a retest visit performed 3 months after the last experimental visit. Individual maximal cardiac frequency (FcMax) was assessed during the VO2max visit. All experimental visits started at 9AM and included two MRI sessions (encoding and test) separated by a physical exercise or rest session. Physical exercise was either of moderate intensity (30 min cycling at 60% FcMax) or of high intensity (15 min cycling at 75% FcMax). Blood samples were collected twice in each experimental visit, before and after exercise or rest. PVT and POMS questionnaire were administered after exercise or rest. (**B**) Examples of series of pictures for each theme: office, shoe shop, or house. (**C**) Examples of direct trials, inference of order 1, and order 2 trials. Direct trials were used during the learning, test, and retest sessions, inferences 1 and 2 trials were used during test and retest sessions. (**D**) Example of control trials. All the presented images were downloaded from the Flickr Creative Commons repository without restrictions to reuse or modify under CC-BY open-access license (https://www.flickr.com/).

We tested 18 participants using a cross-over randomized within-subjects design. We used a hippocampus-dependent associative memory task ^24,29^ in which participants learned 8 series of 6 successive pictures. The participants first saw the eight series once during an encoding session (Fig. 1B), immediately followed by a 2-alternative forced choice learning session with feedback during which participants successively selected the next picture in the series among two presented pictures (Fig. 1C, right panel). To assess the influence of different intensities of physical exercise, we tested the memory for these series following a moderate intensity exercise session, a high intensity exercise session, and a rest period (randomized order of conditions across participants). Blood samples were taken before and after each period of exercise or rest. After exercise or rest, participants were tested on their memory for the associative task on pairs of pictures with different relational distances (direct, inference of order 1 and order 2), with respect to their position in the original series of pictures encoded before the exercise or rest period. Specifically, they were presented with one cue picture, and were then asked which among two target pictures was part of the same series as the cue picture (while the other target picture belonged to a different series) (Fig. 1C). Sixteen control trials were also included, in which the depicted elements were of a given color (red, blue or green) and participants had to choose, between two pictures, the one of the same color as the cue picture. Functional MRI (fMRI) data was acquired during all experimental sessions and analyzed using SPM12 (see “Methods”). We also tested the effects of acute physical exercise on long-term memory during a surprise memory retest, which took place 3 months after the last experimental visit (see Fig. 1A). Our hypothesis is that a single session of physical exercise induces plasticity mechanisms possibly linked to AEA and BDNF increases, which are known to induce LTP or similar mechanisms in the hippocampus. Such changes may promote memory consolidation mechanisms during the exercise session, which would yield better memory performance at test, and possibly more stable memory traces at long-term retest. Further, we expect that, if exercise-related consolidation mechanisms induce lasting synaptic modifications, consolidated memories should be better remembered/or less forgotten when tested after a longer delay. As mentioned above, we hypothesized that moderate intensity exercise would yield the largest memory benefits (especially at immediate test)^24^, and expected that such benefits would be associated with exercise-related changes in BDNF and AEA levels. AEA is known to have transient effects due to its rapid degradation by metabolic enzymes^30^ and has been reported^31^ to have measurable effects on BOLD signal. On the other hand, because it has been previously suggested that BDNF is involved in pattern separation^32^, we predicted that a decoding approach may best capture any possible effect of BDNF changes on memory representations. Finally, based on the reported long-lasting influence of BDNF on exercise-related changes in the hippocampus^4^, we predicted that increases in BDNF levels may underlie long-term memory effects.

## Methods

### Sample size

Because our first aim was to obtain exercise-induced memory improvement (for the moderate exercising condition), we estimated the required sample size based on the results from a previous study, in which the same associative memory task with a similar within-subjects design was used and obtained an effect of exercise on memory performance (14 participants, F(1,13) = 17.27, p = 0.001)^24^. Using the effect size from that previous study (Cohen’s d = 0.53) with an alpha level at 0.05 and power (1-beta) of 0.80, we derived an overall sample size of 15 for a two-tailed dependent-sample t test and we recruited twenty participants.

### Participants

This study was approved by the Ethics Committee of the Geneva University Hospitals**,** all methods were carried out in accordance with relevant guidelines and regulations (corresponding to the Declaration Of Helsinki)**.** Twenty healthy young male volunteers gave written informed consent and received financial compensation for their participation. Participants were all male, right-handed, without psychiatric and neurological history, and reported exercising regularly (at least twice per week). Before inclusion, all participants filled out a sport questionnaire assessing their exercising habits, which we also further clarified during an interview. All participants wore a Fitbit fitness tracker for 5 days before each experimental visit. Thus, physical activity and cardiac frequency was recorded continuously throughout the 5 days. Participants were requested to carry on with their training habits during these 5 days, and to refrain from any strenuous exercise the day before the visit to the lab. We checked compliance with these instructions on the fitness tracker. We could thus ensure that participants maintained their habitual training regime, and that none of them performed excessive exercise the day prior to the experimental measurements. On each experimental day, participants came to the lab at 8 AM on an empty stomach, using public transportation only. We checked each experimental morning that they indeed complied with these requirements. In particular, if they would have cycled in or performed any other sport activity, this would be visible on the cardiac frequency measurement of the Fitbit fitness tracker.

We only included participants whose VO_2max_ was above 40 ml/kg/min and below 65 ml/kg/min (Fig. S4 and Supplementary Information). Two participants had to be excluded from all the analysis for non-compliance with experimental requirements. The remaining 18 participants were between 18 and 34 years old (mean age ± standard error: 23.03 ± 0.92 years).

### Experimental procedure

We assessed the influence of different intensities of physical exercise on memory, by comparing performance during three separate visits including a period of moderate intensity exercise, high intensity exercise, and rest (cross-over randomized within-subjects design). Effort load was calibrated individually, based on the VO_2max_ measure (Fig. 1A). We used a hippocampus-dependent associative memory task, in which participants learned 8 series of 6 successive pictures^24,29^. To avoid interference across experimental visits for this within-subjects design, participants learned series of pictures belonging to different themes at each of the three visits: “office”, “shoe shop” or “house” (one theme per visit). The pictures from the 8 series presented belonged to the same category. For example, each the “office” theme series was composed of a pen, a chair, a desk, an office space, a meeting room, and a building, with 8 different exemplars of those pictures for the 8 series. The pictures in each theme for the experimental visits were matched in difficulty and counterbalanced across Exercising conditions and visits (Fig. 1B). Over the 5 days prior to each experimental visit, participants wore a Fitbit fitness tracker (Fitbit Charge 3HR) to monitor their exercising habits and to ensure that they did not engage in intense workouts during the day preceding the experiment. On each experimental day, participants came to the lab in the morning on an empty stomach and had breakfast with us (Supplementary Information for details). They entered the MRI scanner at 9AM, they saw all 8 series once during an encoding session (Fig. 1B), followed by a 2-alternative forced choice (2AFC) learning session with feedback (Fig. 1C). At 09:50 AM a qualified medical doctor took a first blood sample. At 10:00 AM participants were equipped with a Polar RS800CX N device to measure heart rate and asked to rest or exercise. For moderate intensity exercise, each participant pedaled for 30 min at a cardiac frequency of 65% of their FcMax. For high intensity, participants warmed up for 2 min at 50% FcMax then the load was progressively increased over 1 min to reach 75% FcMax. Participants pedaled at this intensity for 15 min then they pedaled at 50% FcMax for 3 min to cool down. For the rest condition, participants sat on a chair and were allowed to quietly look at magazines for 30 min. At 10:30 AM, the medical doctor took a second blood sample and 15 min later, participants performed a Psychomotor Vigilance Task (PVT) followed by the Profile of Mood States (POMS) questionnaire. At 11:30 AM, participants underwent a second fMRI session during which memory for the associative task was tested using a 2AFC on pairs of pictures with different relational distances (direct, inference of order 1 and order 2; Fig. 1C). Sixteen control trials (used in the decoding analysis, see below) were also included, in which the depicted elements were of a given color (red, blue or green) and participants had to choose among two pictures which one was of the same color as the target picture. A surprise retest fMRI session took place 3 months later where participant’s memory was tested again; no blood samples were taken at this time point (Fig. 1A). See Supplementary Information for VO2max procedure and further details on the experimental procedure.

#### Behavioral analysis

We analyzed memory accuracy measures (% of correct responses). Moreover, because the existing literature suggests that physical exercise may influence discrimination times as in the Stroop task^33^, we also analyzed efficiency measures (i.e. accuracy measures divided by reaction times) to minimize any potential speed-accuracy trade-off effects. We used repeated-measures ANOVAs and Newman–Keuls post-hoc comparisons. Correlation analyses were performed using Spearman’s Rho. All behavioral analyses were conducted using Statistica12 (StatSoft, Inc. TULSA, OK, USA).

#### Functional MRI (fMRI) data acquisition

MRI data were acquired on a 3 Tesla MRI scanner (SIEMENS Trio^®^ System, Siemens, Erlangen, Germany) with a 32-channel head coil, see Supplementary Information for details.

#### fMRI analysis

Functional images were analyzed using SPM12 (Wellcome Department of Imaging Neuroscience, London, UK) using standard preprocessing procedures. A general linear model (GLM) approach was then used to compare conditions of interest at the individual level. Each individual GLM included correct trials separated according to Relational distance (direct, inference 1, inference 2), control trials and incorrect trials (pooled across Relational distance). Additionally, 6 movement regressors, 5 heart rate regressors and 1 breathing regressor (to correct for potential heart- and breathing-related artifacts^34,35^, see Supplementary Information) were added as regressors of non-interest. Then, contrasts between conditions of interest from each participant were entered a second-level random-effects analysis. We report activations at 0.001 uncorrected, with a minimal cluster size of 10 contiguous voxels that survive small volume correction for hippocampal activations. We also applied a decoding approach to further characterize how exercise modulated the strength of hippocampal memory representations as expressed by voxel-based activation patterns (see Supplementary Information).

## Results

### Learning

Hit rate and efficiency (i.e. hit rate divided by reaction time) were analyzed using repeated measure ANOVAs with Learning blocks (block 1, block 2, block 3) and Visit theme (office, shoe shop, house) as within-subjects factors. Both analyses revealed a main effect of Learning block (hit rate: F(2, 34) = 23.16, p < 0.001; efficiency: F(2, 34) = 17.64, p < 0.001), consistent with a progressive learning of the associations, but no effect of Visit theme (hit rate: F(2, 34) = 1.92, p = 0.16; efficiency: F(2, 34) = 0.16, p = 0.85) and no interaction (hit rate: F(4, 68) = 0.56, p = 0.69; efficiency: F(4, 68) = 0.41, p = 0.80). Importantly, there was no main effect and no interaction with subsequent physical exercise neither for hit rate nor for efficiency (all p > 0.05) when this factor was added as repeated measure to the previous ANOVAs. Overall, during block 3, participants reached a high level of performance (hit rate ± standard error: 86.94 ± 1.63%), suggesting a good encoding of the series, well above chance level. All statistical results are reported in Table 1. Although brain imaging data were acquired during learning, we do not report them here as this session simply constitutes a baseline before our physical exercising sessions.

**Table 1:** Statistical results. Significant effects are highlighted in bold.

ANOVAS	Result	Neuman–Keuls post-hoc tests
Learning: accuracy (% correct)	**Block: F(2, 34) = 23.16, p < 0.00001** Visit theme: F(2, 34) = 1.92, p = 0.16 Interaction: F(4, 68) = 0.56; p = 0.69	**Block 1–2 p = 0.008** **Block 1- p = 0.0001** **Block 2–3: p = 0.0005**
Learning: efficiency (accuracy/reaction time (s))	**Block: F(2, 34) = 17.64, p = 0.00001** Visit theme: F(2, 34) = 0.16, p = 0.85 Interaction: F(4, 68) = 0.41, p = 0.80	**Block1-2 p = 0.002** **Block 1–3 p = 0.0001** **Block 2–3 p = 0.016**
Heart rate	**Exercising condition: F(2, 34) = 1672.9, p < 0.00001**	**Mod-rest p = 0.0001** **High-rest p = 0.0001** **Mod-high p = 0.0001**
Test: accuracy For visit theme and relational distance	Visit theme: F(2,102) = 1.40, p = 0.25 Relational distance: F(2,51) = 0.09, p = 0.90 Interaction: F(4,102) = 0.28, p = 0.89	
Test: accuracy	**Exercising condition: F(2, 102) = 4.01, p = 0.02** Relational distance: F(2,51) = 0.14, p = 0.98 Interaction: F(4,102) = 0.45, p = 0.77	**Mod-rest p = 0.02** High-rest p = 0.33 Mod-high p = 0.07 (trend)
Test: efficiency	**Exercising condition: F(1, 102) = 6.03, p = 0.003** Relational distance: F(2,51) = 0.30, p = 0.74 Interaction: F(4,102) = 0.043, p = 0.99	**Mod-rest p = 0.016** High-rest p = 0.37 **Mod-high p = 0.0032**
AEA	**Exercising condition: F(2, 34) = 39.27, p < 0.00001**	**Mod-rest p = 0.0001** **High-rest p = 0.0001** Mod-high p = 0.12
BDNF	**Exercising condition: F(2, 34) = 4.78, p = 0.01**	**Mod-rest p = 0.045** **High-rest p = 0.01** Mod-high p = 0.17
PVT	Mean, median reaction times, nb lapses, nb false alarms: all p > 0.05 for Exercising condition	
POMS	Exercising condition: all p > 0.05	
Decoding	**Trial Type: F(2, 34) = 33.77, p < 0.00001** Exercising condition: F(2,35) = 2.03, p = 0.15 **Interaction: F(4, 68) = 2.58, p = 0.04**	**Hit-error p = 0.0001** **Hit-control p = 0.02** **Error-control p = 0.0001**
Decoding of hits	**Exercising condition: F(2, 34) = 9.43, p = 0.0006**	**Mod-rest p = 0.0001** High-rest p = 0.66 **Mod-high p = 0.0001**
Decoding of errors	Exercising condition: F(2,34) = 0.24, p = 0.78	
Decoding of control trials	Exercising condition: F(2,34) = 2.28, p = 0.12	
Retest: accuracy	Exercising condition: F(2,34) = 3.46, p = 0.042	**Mod-rest p = 0.03** High-rest p = 0.18 Mod-high p = 0.21
Retest: efficiency	Exercising condition: F(2,34) = 2.93, p = 0.07	
**T-TESTS**		**Result**
Retest Moderate vs chance level		**t(17) = 2.81, p = 0.01**
Retest Rest vs chance level		t(17) = − 1.28, p = 0.21
Retest High vs chance level		t(17) = 0.51, p = 0.62
**CORRELATIONS**		
VO2max vs. hit rate, efficiency, AEA, BDNF		All p > 0.05
**ΔBDNF mod-rest vs decoding moderate** ΔAEA mod-rest vs decoding moderate		**R = 0.53, p = 0.02, p**_**corrected**_** = 0.04** R = − 0.03, p = 0.889
ΔBDNF high-rest vs decoding high ΔAEA high-rest vs decoding high		R = 0.21, p = 0.13, p_corrected_ = 0.26 R = − 0.11, p = 0.66
**ΔBDNF mod-rest vs performance test to retest moderate** ΔAEA mod-rest vs performance test to retest moderate		**R = 0.55, p = 0.02, p**_**corrected**_** = 0.04** R = 0.177, p = 0.48
ΔBDNF high-rest vs performance test to retest high ΔAEA high-rest vs performance test to retest high		R = 0.31, p = 0.20, p_corrected_ = 0.40 R = 0.01, p = 0.96

### Heart rate and breathing analysis

Participants’ heart rate, measured by a Polar cardiofrequencemeter (Polar RS 800 CX, Polar, Finland), during the rest period was at 33.4 ± 4.0% of their maximal heart rate as assessed by the VO2max procedure (see “Methods”). During moderate and high intensity physical exercise, participants pedaled at 68.7 ± 1.1% and 77.7 ± 1.9% of their maximal heart rate, respectively. A one way repeated-measures ANOVA revealed a significant difference in heart rate between the three Exercising conditions (rest, moderate, high intensity exercise; F(2, 34) = 1672.9, p < 0.001). Post-hoc analyses confirmed that the three Exercising conditions differed from each other (all p < 0.001). We also recorded heart rate during the test part in fMRI using the Biopac and found no difference in heart rate as a function of the Exercising condition (all p > 0.05), suggesting that all participants’ heart rates were back to baseline at test (i.e., at least 45 min after the completion of the exercise session).

### Psychomotor vigilance test (PVT) and Profile of Mood States questionnaire (POMS)

We administered the PVT and POMS just before the MRI test session (i.e. about 45 min after rest or exercise) to monitor possible condition-dependent differences in vigilance and mood at the time of the test session^36^. This was more than half-an-hour after the end of the exercising/rest period when heart rate and other physiological measures were back to baseline. For PVT, similarly to previous findings^24^, we show no difference in PVT as a function of Exercising condition (rest, moderate, high), neither in mean or median reaction times, number of lapses, or number of false alarms (one way repeated measures ANOVAs, all p > 0.05). This suggested that participants did not significantly differ in vigilance state 45 min after rest or physical exercise. Concerning the POMS questionnaire, we used a short version composed of 38 questions assessing tension, fatigue, vigor, confusion with 7–9 questions for each category and including an additional 7 dummy questions. Each question has 5 levels: 0 = not at all, 1 = a little, 2 = moderately, 3 = quite a lot, 4 = extremely. The POMS score for each category is the sum of the scores for the corresponding questions. For POMS, we report no difference for any of the measured categories (fatigue, tension, confusion, vigor) as a function of Exercising condition (all p > 0.05), suggesting that the physical exercise sessions did not result in lasting mood changes.

### Test

#### Behavior

**Figure 2:**
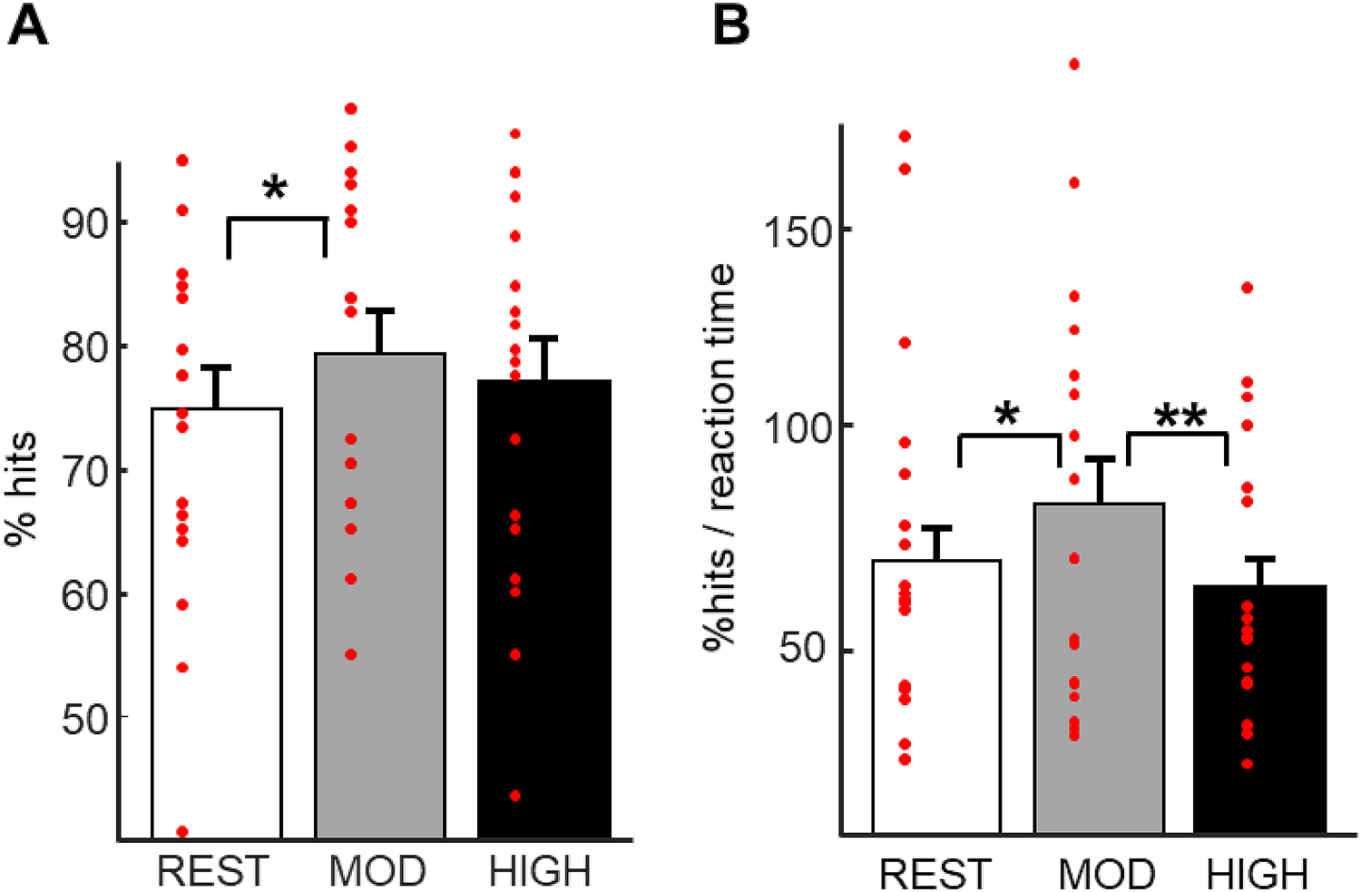
Memory performance at test. (**A**) Accuracy: higher percentage of hits after moderate intensity exercise than after rest. (**B**) Efficiency (% hits/reaction time (s)): higher efficiency after moderate intensity exercise than after rest and high intensity exercise. Error bars represent SEM.

To test our main prediction about the effect of physical exercise on memory and provide a replication of previous behavioral findings^24^, accuracy and efficiency data from the test session were analyzed using repeated-measures ANOVAs with Exercising condition (rest, moderate intensity exercise, high intensity exercise) and Relational distance (direct, inference 1, inference 2) as within-subjects factors. We report a main effect of Exercising condition for both accuracy (F(2, 102) = 4.01, p = 0.02) and efficiency (F(1, 102) = 6.03, p = 0.003), but no effect of Relational distance (p = 0.74) and no interaction (p = 0.99; see Fig. 2; Table 1). Post-hoc analyses revealed higher accuracy and efficiency after the moderate compared the rest Exercising condition (p_mod-rest acc_ = 0.02 and p_mod-rest eff_ = 0.016, respectively), while efficiency was also higher after moderate intensity exercise compared to high intensity exercise (p_mod-high eff_ = 0.0032).

#### Reaction times

Although we did not ask participants to answer as quickly as possible, the reaction times clearly varied as a function of the Response-type (hits, misses; see figure below). A repeated-measure ANOVA Response-type by Exercising condition showed a main effect of Response-type with faster responses for hits than for misses (F(1, 17) = 41.118, p < 0.001, 1432 ms ± 627 ms for correct hits; 2124 ms ± 676 ms for errors), but no effect of Exercising condition, and no interaction (all p < 0.05).

#### Correlation with general fitness level

To investigate whether individual fitness level may have had a modulatory effect on performance, we first performed exploratory correlations between behavioral measures (hit rate and efficiency for each Exercising condition) and VO2max measures, but found no correlation (hit rate: p_rest_ = 0.304, p_mod_ = 0.835, p_high_ = 0.090; efficiency: p_rest_ = 0.639, p_mod_ = 0.615, p_high_ = 0.275). Then, to test whether individual VO2max may have affected the relationship between exercise and memory performance, we performed correlation analyses between VO2max and memory benefits (from rest to moderate or high exercise). We did not find any significant correlation, although there was a non-significant trend for a correlation between hit rate improvement after moderate intensity exercise (vs. rest) and VO2max (hit rate: p_mod-rest_ = 0.085 and p_high-rest_ = 0.595; efficiency: p_mod-rest_ = 0.329 and p_high-rest_ = 0.701).

#### Visit theme

Additionally, we tested for possible effects of Visit theme (see “Methods”). We therefore performed a repeated-measures ANOVA with Visit theme (office, shoe shop, house) and Relational distance (direct, inference 1, inference 2) as within-subjects factors. We report no effect of Visit theme (F(2, 102) = 1.40, p = 0.25) and no effect of Relational distance and no interaction effect (both p > 0.05).

#### Blood samples

**Figure 3:**
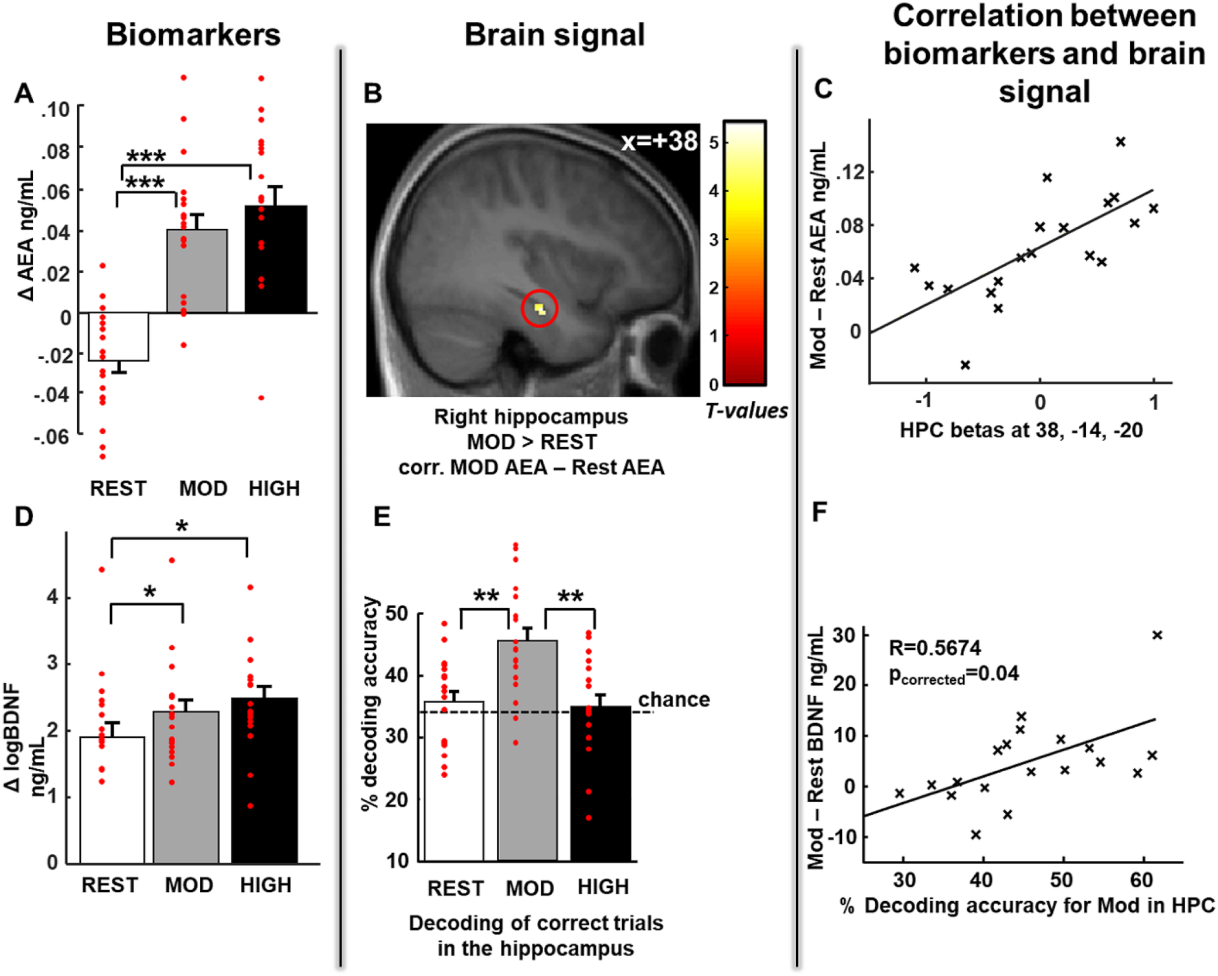
Increased biomarker levels correlate with hippocampal brain signals after moderate intensity exercise. (**A**) Increased Anandamide level (AEA) after moderate and high physical exercise compared to rest. For all Exercising Conditions Δ AEA corresponds to the difference in AEA between the second blood sample (after exercise or rest) and first blood sample (before exercise or rest). (**B**) Increased right hippocampal activity for hits after moderate exercise compared to hits after rest correlated with the increase in AEA level after moderate exercise [z score = 3.72 (38, − 14, − 20), p < 0.05 SVC corrected for multiple comparisons]. (**C**) Correlation between the beta values from the right hippocampal activation described in (**B**) and AEA, shown here for illustrative purposes only. (**D**) Increased BDNF levels after moderate and high intensity exercise compared to after rest. For all Exercising conditions, Δ BDNF corresponds to the difference in BDNF between the second blood sample and the first blood sample. (**E**) Higher decoding accuracy for hits in the bilateral hippocampus after moderate exercise than after rest and high intensity exercise. (**F**) Significant positive correlation between decoding accuracy in the hippocampus and increase in BDNF level after moderate intensity exercise (R = 0.53, p = 0.04, corrected for multiple comparisons). Activation map displayed on the mean T1 anatomical scan of the studied population.

For each visit, we measured changes (i.e. differences) in endocannabinoids and BDNF levels from the blood samples collected before (baseline value) and after the rest or exercise sessions. Repeated-measures ANOVAs were performed for each biomarker with Exercising condition (rest, moderate, high) as a within-subjects factor. For AEA, a main effect of Exercising condition (F(2, 34) = 39.25, p < 0.00001; Fig. 3A) was found. Post-hoc analyses revealed that AEA levels were lower after rest than after physical exercise (p_rest-mod_ = 0.0001, p_rest-high_ = 0.00001), with no difference in AEA levels between moderate and high intensity exercise (p_mod-high_ = 0.12). For BDNF, we report a main effect of Exercising condition (F(2, 34) = 4.78, p = 0.01; Fig. 3D). As for AEA above, post-hoc analyses revealed that, BDNF levels after moderate and high intensity exercise differed from after rest (p_rest-mod_ = 0.045, p_rest-high_ = 0.01). For the endocannabinoid 2-arachidonoylglycerol (2-AG), there was no effect of Exercising condition (F(2, 34) = 2.90, p > 0.05), consistent with previous descriptions in the literature^14^.

#### fMRI

Based on the observation that AEA has a rapid effect on synaptic plasticity in the hippocampus, we expected that changes in AEA levels across Exercising conditions might exert a modulatory influence on brain activity. We therefore included AEA change as a cofactor in the second-level analyses comparing Exercising conditions. We specifically tested for AEA because of its fast synthesis and metabolism rates which, at the timescale of a few hours, are highly compatible with rapid plasticity changes. Yet, because BDNF might also have a modulatory effect on hippocampal activity, we also performed the same analysis with BDNF, and consequently corrected our results for multiple testing using Bonferroni methods. We found that activity in the right hippocampus for hits correlated with the increase in AEA after moderate intensity exercise (vs. rest) [z score = 3.72 (38, − 14, − 20), p < 0.05 SVC, corrected for multiple testing] (Fig. 3B,C and Table 2), and after high intensity exercise (vs. rest) [z score = 4.08 (32, − 24, − 18), p < 0.05 SVC] (Fig. S2). To assess whether a similar relationship may also be present for BDNF, we first performed a correlation analysis between the extracted hippocampal beta values and the changes in BDNF, and found no significant correlation (R = 0.21, p = 0.40).We then compared the strength of the correlation between hippocampal univariate activity and AEA and BDNF, respectively, and found that the correlation with AEA was significantly stronger than the correlation with BDNF (Fisher’s R to Z = 1.743, p = 0.04), confirming that univariate hippocampal activity has a strong and selective linear relationship with AEA.

**Table 2:** Activated brain regions at test.

Brain region	Lat	Cluster size	Unc. p value	SVC p value	Peak T	Peak Z	X	Y	Z
**Increasing relational distance (inference 2 hits > direct hits)**									
Precuneus	Right	579	7.9E−07		7.16	4.80	16	− 46	14
Precuneus	Left	366	8.4E−06		5.92	4.30	− 18	− 54	− 10
Hippocampus	Right	190	4.2E−06¤	0.02	6.27	4.35	18	− 38	− 8
Subiculum	Right	15	3.7E−04¤	0.01	4.10	3.37	26	− 28	− 20
Lingual gyrus	Right	50	3.6E−05		5.20	3.97	16	− 82	− 6
Occipital gyrus	Right	13	4.9E−04		3.97	3.29	− 12	− 92	0
**Moderate intensity exercise > rest corr. with changes in AEA**									
Hippocampus	Right	13	1E−04¤	0.03 (corrected for multiple testing)	5.11	3.72	38	− 14	− 20
**High intensity exercise > rest corr. with changes in AEA**									
Parahippocampus	Left	35	9.4E−6		6.54	4.28	− 30	− 28	− 18
Parahippocampus	Right	29	2.7E−5		5.99	4.08	34	− 24	− 22
Hippocampus (extending from parahippocampus above)	Right	29	2.7E−5¤	0.014 (corrected for multiple testing)	4.95		32	− 24	− 18
Middle occipital gyrus	Right	21	6.5E−05		5.36	3.83	42	− 78	36

Activations in the hippocampal formation corrected using an anatomical mask from Anatomy toolbox (see “Methods”).

When comparing high Relational distance to low Relational distance (inference 2 > direct trials) across all sessions, we found increased activity in the right hippocampus [z score = 4.35 (18, − 38, − 8), p < 0.05 SVC, see “Methods”], bilateral parahippocampal gyrus and precuneus; see Fig. S1 and Table 2 for an exhaustive list of activated regions. These regions overall correspond to previous findings^29,37^. No region was activated (at a threshold of 0.001 unc.) when comparing inference 1 to direct trials, and inference 2 to inference 1 trials. To assess for the main effects of exercising condition, we conducted a standard general linear model analysis with the data collected during the memory test after rest, moderate intensity exercise, and high intensity exercise modelled as separate sessions. Within each session, we considered hits according to Relational distance (direct, inference 1, inference 2) and control trials as four separate regressors of interest, and included missed trials as an additional regressor (Fig. 1). Comparisons between Exercising conditions and interactions between Relational distance and Exercising conditions did not yield any significant activation either.

Next, we applied a decoding approach to test whether exercise would affect the coherence of the fine-grained neural representation of hits, errors and control trials within the bilateral hippocampus. Using a similar procedure as in Van Dongen et al.^22^, we classified voxelwise hippocampal activity patterns from each trial, as belonging to one of three possible outcomes (hits, errors or control trials), with a chance level at 33.33%. We performed an ANOVA on classification accuracy with Trial Type (hits, errors, or control trials) and Exercising condition (rest, moderate, high) as factors. We observed an effect of Trial Type (F(2, 34) = 33.77, p < 0.001) and an interaction between Exercising condition and Trial Type (F(4, 68) = 2.58, p = 0.04). Separate analyses for each trial type showed that hits were better classified in the moderate condition (F(2, 34) = 9.43, p < 0.001), while there was no effect of Exercising condition for the control trials and errors (p = 0.12 and p = 0.78 respectively). We performed an ANOVA on classification accuracy with Trial Type (hits, errors, or control trials) and Exercising condition (rest, moderate, high) as factors. We observed an effect of Trial Type (F(2, 34) = 33.77, p < 0.001) and an interaction between Exercising condition and Trial Type (F(4, 68) = 2.58, p = 0.04). Separate analyses for each trial type showed that hits were better classified in the moderate condition (F(2, 34) = 9.43, p < 0.001), while there was no effect of Exercising condition for the control trials and errors (p = 0.12 and p = 0.78, respectively). Therefore, we focused on hits only for the next analyses, we report that decoding accuracy for hits was above chance level after moderate intensity exercise (t(17) = 2.81, p = 0.01), but at chance level after rest and high intensity exercise ((t_rest_(17) = -1.28, p = 0.21, t_high_(17) = 0.51, p = 0.62, Fig. 3E). Post-hoc analyses showed that decoding after moderate intensity exercise was higher than after both rest and high intensity exercise (F(2,34) = 9.43, p = 0.0006, p_mod-rest_ = 0.0001, p_mod-high_ = 0.0001, whereas p_high-rest_ = 0.66 Fig. 3E). We obtained similar results when we performed the classification on activity from the left or the right hippocampus separately (Fig. S3). Our results show that, after moderate intensity exercise, the percentage of correctly classified trials for hits was above chance, whereas after rest and high intensity exercise this percentage was at chance level. Percentages of correctly classified errors and control trials did not differ from chance for any exercise condition. These results may suggest that moderate intensity exercise benefited memory consolidation processes by promoting the emergence of stable hippocampal activation patterns. Increased decoding accuracy in the hippocampus and the medial temporal lobe for memory tasks has previously been proposed to predict source memory performance^38,39^. This mechanism could also underlie some confidence effects since better remembered information in a similar task has been shown to be associated with higher confidence^29^.

Because BDNF is known to specifically enhance synaptic plasticity mechanisms in the hippocampus, we tested whether BDNF levels affected hippocampal neural representations as estimated by the decoding results. As AEA could also contribute to neural plasticity, we performed the same analysis with this molecule, and corrected both analyses for multiple testing using Bonferroni correction. We report a positive correlation between BDNF enhancement during moderate intensity exercise (calculated as the difference between moderate and rest BDNF changes) with decoding accuracy after moderate intensity exercise (R = 0.53, p = 0.04, corrected for multiple testing, Fig. 3F). No such correlation was found with AEA (R = − 0.03, p = 0.889, see also Table 1). To assess the selectivity of the relationship between decoding and BDNF, we compared the strength of the correlations between decoding accuracy and AEA and BDNF, respectively, and obtained a significant difference in correlation (Fisher’s R to Z = 1.853, p = 0.032).

### Retest

**Figure 4:**
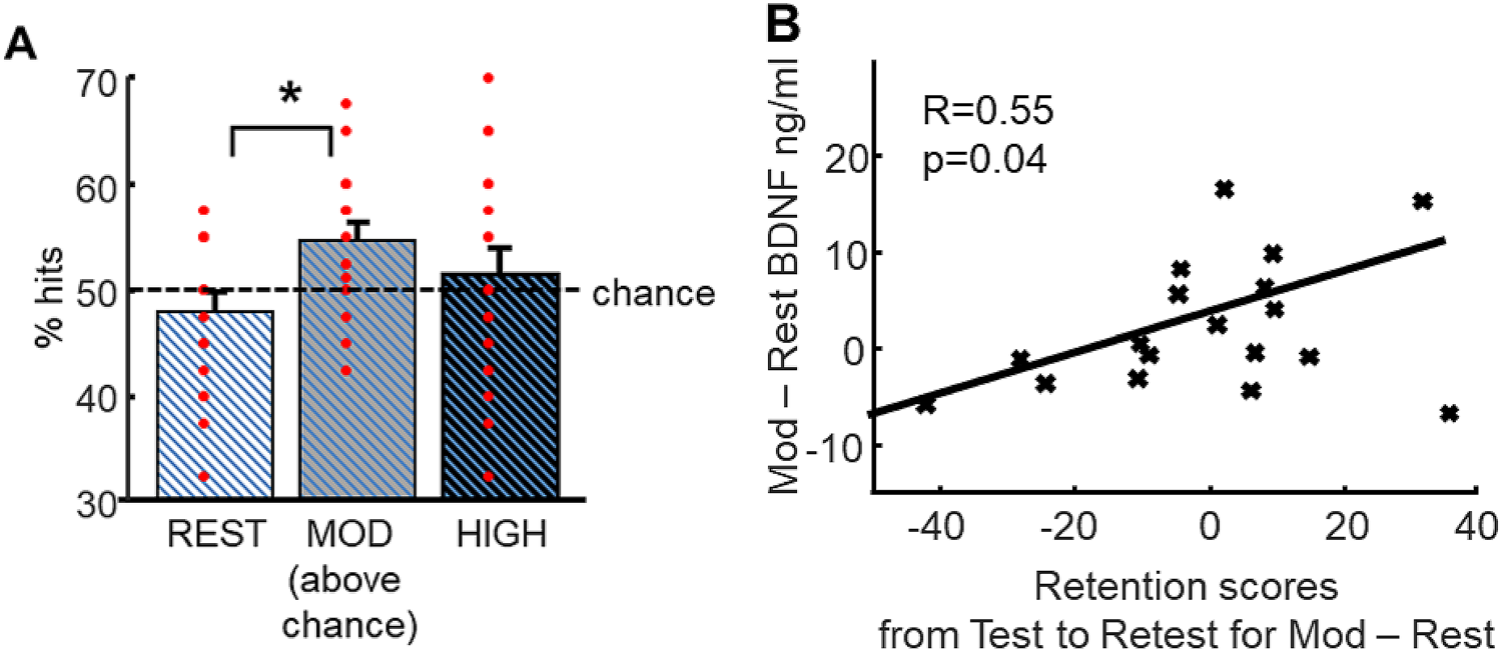
Better 3-month long-term memory for associations learned after moderate physical exercise and link with BDNF. (**A**) Better performance for pictures learned during the moderate intensity visit than for pictures learned during the resting visit. Performance after moderate exercise was significantly above chance level. (**B**) Retention scores from test to retest for moderate exercise compared to rest significantly correlated with BDNF enhancement from moderate exercise to rest (R = 0.55, p = 0.04, corrected for multiple comparisons).

All participants came back for a long-term memory retest session 3 months later. Retest was similar to the test sessions but it comprised five trials for each of the eight sequences of pictures of all three themes (24 sequences) amounting to 120 trials. For each sequence, two direct trials, two inference order 1 trials and one inference order 2 trial were included to equalize difficulty from all visits. For the analysis of the retest session, trials were not separated according to Relational distance as no behavioral effect related to this was previously found. A repeated-measure ANOVA conducted on accuracy and efficiency measures with Exercising condition (rest, moderate, high) as within-subjects factor revealed a main effect of Exercising condition for accuracy (F(2, 34) = 3.46, p = 0.042) and a tendency for efficiency (F(2, 34) = 2.93, p = 0.07). For accuracy, post-hoc analyses showed that participants performed better for associations learned during the moderate condition as compared to those learned during rest condition (p_mod-rest_ = 0.03; Fig. 4A). Furthermore, only the trials from the moderate exercise session were remembered above chance (t(17) = 2.81, p = 0.01).

Because BDNF contributes to neurogenesis and synaptic plasticity^4,7,8^, we hypothesized that changes in BDNF levels during the moderate intensity exercise condition may potentially promote long-term memory retention. We therefore correlated individual changes in BDNF levels for moderate intensity (vs. rest) to delayed memory retention (from test to retest) for items initially learned during the moderate (vs. rest) condition. As AEA might also have a plasticity effect, we subsequently performed the same analysis with AEA, and corrected both results for multiple testing using Bonferroni correction. We report a significant positive correlation between BDNF and delayed memory retention (R = 0.55, p = 0.04, corrected for multiple testing; Fig. 4B), and no effect for AEA (R = 0.177, p = 0.48). Yet, a direct comparison of the strength of these correlations did not yield a significant difference (Fisher’s R to Z = 1.20, p = 0.11), thus suggesting that we cannot conclude that correlation between BDNF and delayed memory retention was stronger than between AEA and delayed memory retention. Please note that the same correlation for changes in BDNF after high intensity exercise was not significant (R = 0.31, p = 0.20).

## Discussion

Here we show that a single session of moderate intensity physical exercise compared to a period of rest enhanced associative memory, both at immediate test (2 h after encoding) and at long-term retest (3 months later). These effects may be linked to the endocannabinoid AEA and the growth-factor BDNF, whose respective concentrations increased after acute exercise. Accordingly, during the short-term test, the increase in plasma AEA concentration correlated with hippocampal activity when associative memories were recalled, and BDNF increase correlated with decoding measures within the hippocampus. Moreover, BDNF increase during moderate intensity physical exercise correlated with better performance at long-term retest. We did not observe such memory benefits for a session of high intensity physical exercise, i.e. when effort levels surpassed the ventilatory threshold. Overall, we show that acute physical exercise at moderate intensity has long-lasting positive effects on the consolidation of associative memories in healthy young human adults. Below, we discuss the neurophysiological mechanisms that could explain these findings.

### Neuromodulatory mechanisms underlying the effects of acute exercise on hippocampal plasticity

A recent study in rodents^40^ demonstrated that acute physical exercise induces a transient increase of AEA measured in the plasma, with direct effects on CB1 receptors in the brain. Note that in the same study cerebro-spinal fluid measures did not capture increases in AEA, consistent with AEA being very rapidly metabolized in the brain^30^. These results support the fact that plasma measures of AEA, as we performed here, offer a reliable index of AEA activity in the central nervous system. Interesting animal data further hints that activating CB1 receptors can be measured in univariate BOLD activity^31^ which is similar to the correlational measure we observe here.

Another rodent study directly linked endocannabinoid signaling to hippocampal memory function by showing that selectively blocking CB1 receptors in the hippocampus abolished exercise-induced memory effects^21^. Based on these rodent studies, we suggest that similar neurophysiological mechanisms may account for our novel finding that exercise-induced AEA increase in human plasma related to brain activity, especially in the hippocampus, during successful memory recall.

Consistent with our findings, the existing literature on AEA suggests that AEA levels are highest for exercise levels around 72% of maximal age-adjusted theoretical heart rate, and drop at 83% and 92% of maximal heart rate^15^. In our study, the moderate intensity would correspond to 66.77 ± 2.23% and the high intensity to 75.48 ± 2.79% of maximal age-adjusted theoretical heart rate, thus both corresponding to the exercising zone around the maximal increase in AEA, but with high intensity exercise close to the turning point after which AEA levels drop. Because the intensity at which AEA peak is reached may vary as a function of individual fitness level, metabolism rate, and stress levels induced by exercise^41^, we would expect that moderate intensity exercise in our study may elicit a more systematic AEA increase across individual than the high intensity exercise. These factors may have contributed to the increased inter-individual variability in biomarker levels/results after high intensity condition. Conversely, moderate intensity exercise would thus confer an optimal and stable boost in biomarkers, which may robustly relate to enhanced long term memory performance.

Also consistent with our results, recent findings emphasized that light to moderate intensity exercise has an effect on memory^24^ and hippocampal pattern separation^25^, especially for one uninterrupted bout of exercise at a given intensity as we describe here (sometimes referred to as endurance exercise as opposed to interval training comprised of several very short epochs of exercise).

Conversely, previous results suggested that high intensity interval training may benefit memory only if exercise occurred 4 h after learning and when tested 48 h after encoding^22^. Here, using distinct levels of exercise in the same individuals, we establish that, for endurance exercise, moderate exercise intensity represents an optimal condition for memory performance.

Traditionally, BDNF has been linked to effects of regular physical exercise, although it is known that BDNF gene expression is upregulated after both acute and regular physical exercise in rodents^42^. It is widely acknowledged that BDNF enhances synaptic plasticity, especially via LTP^12^, which can be induced in a few minutes and critically contributes to memory consolidation^43^. BDNF plays a fundamental role in triggering protein synthesis in plasticity mechanisms such as LTP maintenance^44^, which is why it would be more likely linked to changes in decoding rather than AEA. This increase in decoding accuracy may reflect increased coherence and efficiency in the retrieval of these memory representations after moderate intensity Exercising condition. Here we show that BDNF increase after one single session of moderate physical exercise may affect both short and long-term memory retention. Specifically, acute BDNF increase correlated positively with decoding accuracy of memory items in both hippocampi immediately after exercise (test session), while it also favored long-term memory retention (at 3-month retest).

### Acute moderate intensity exercise benefits memory consolidation

While characterizing the impact of exercise intensity on cognitive functions is critical for health recommendations, including dementia prevention programs and rehabilitation strategies, the reported effects remain inconsistent. Some studies suggest that high intensity training is most efficient^22,23^, while other studies, among which meta-analyses, indicate that moderate or light intensity exercise might have more impact^25,45^. Here we aimed at clarifying this important issue by using a cross-over randomized within-subjects design according to which each participant was tested at a moderate and at a high intensity (plus a resting, baseline condition) across distinct sessions together with associative memory testing. Importantly, here we determined moderate and high intensity exercise levels with reference to each participant’s individual ventilatory threshold measured using a VO2max procedure, see Svedahl et al. for review^46^. We found strong beneficial effects of moderate intensity exercise on memory, with significant differences compared to rest, while the effects of high intensity exercise appeared to be less robust or absent. Existing evidence has suggested that hippocampal functioning is enhanced by aerobic exercise training (i.e. below ventilatory threshold) but not by high intensity training^47^. One plausible explanation is that acute high intensity exercise may induce a physiological stress response (e.g. elevated cortisol levels), which in turn may impair memory for previously learned stimuli^48,49^. Please note that we observed that both moderate and high exercise intensity increased BDNF and AEA levels, thus further supporting that other factors may yield deleterious effects on performance during high intensity exercise. Although we did not observe that biomarker levels were significantly higher during high compared to moderate exercise, they were numerically higher (Fig. 3A,B). We may thus also tentatively speculate that large increases in BDNF and/or AEA concentrations might not be as beneficial for memory performance. Consistent with this suggestion, intermediate concentrations of BDNF have been reported to increase rodent hippocampal plasticity most^50^. Similarly, for AEA, highest concentrations of AEA were shown to be less effective than intermediate concentrations^51^. The present study would thus offer additional evidence supporting that submaximal concentrations of both molecules (as obtained after moderate intensity exercise) may optimally assist neurocognitive functions, especially hippocampal-dependent memory consolidation.

How can we explain that an acute modulation of BDNF levels affects memory? BDNF facilitates LTP by activating signaling pathways (including MAPK and Akt)^52^, promoting cytoskeleton changes^53^, and enhancing protein synthesis required for vesicle trafficking and the release of neurotransmitters^54^. Several studies have now confirmed that physical exercise, both per se and via BDNF signaling, boosts LTP^12,55^ via glutamatergic NMDA receptor activation. Indeed, on the one hand, physical exercise increases the expression of both NR2A and NR2B subtypes of the NMDA receptor in the hippocampus^11,42^ while, on the other hand, BDNF modulates the activity of NMDA receptors at hippocampal synapses^56^. Studies in rodents have repeatedly shown that increasing NMDA-receptor-mediated plasticity is crucial for associative memory acquisition and consolidation^57,58^. In our experimental design, inducing LTP in the hippocampus (through exercise) after the encoding of new associations likely strengthens memory representations and consolidation, hence affecting pattern completion in the hippocampus for example^59^. Hence supporting better classification results in the Exercising condition that promoted LTP (i.e. moderate exercise).

### Long-term consequences of acute physical exercise on memory consolidation

Lasting effects of physical exercise are established for regular physical exercise protocols, involving several months of training^4,60^. Yet, consistent with the present results at immediate test, acute exercise has also been reported to have positive short-term cognitive effects^22,24,61^. Long-term effects of acute physical exercise (at the scale of several months, as tested here) have to our knowledge not been investigated in humans so far. Here we found that acute physical exercise performed right after memory encoding resulted in both short- and long-lasting improvements of memory retention. We also report long-term effects for moderate intensity exercise, which were associated with increased BDNF levels. Although, we found a significant correlation between performance from test to retest and changes in BDNF concentration after moderate intensity exercise (versus rest), and no such correlation relative to AEA, we cannot conclude that long-term performance had a stronger relationship to BDNF relative to AEA, because the strengths of these correlations did not significantly differ. Even if efficiency results for the long-term retest were marginal, the accuracy results showed a significant lasting effect for the moderate intensity exercise.

### Possible confounding factors due to fatigue or carry-over effects of exercise

Fatigue and reduced vigilance are known to affect cognitive performance. We sought to minimize any potential effect of exercise-related fatigue (1) by scheduling the test part of the protocol 1 h after the end of the physical exercise session and performing all experimental visits at the same time of the day (always in the morning from 8 to 12 AM); (2) by including only participants who were exercising regularly and whose VO2max levels were above 40 ml/kg/min, so that exercise intensity and duration would not be exhausting for them. We also specifically measured fatigue and vigilance levels in our participants and found that neither POMS scores for fatigue nor PVT did differ after moderate or high intensity exercise. We also checked that heart rate and breathing rhythm of all our participants were back to baseline levels when the test session started. Finally, to exclude any contaminations of heart rate or breathing on our fMRI data, we carefully regressed out these effects using Retroicor^34^ and RVHcorr^35,62^.

### Limitations

There are several limitations in this study, among which we here list the most relevant ones. First, we estimated the required sample size based on a previous study using the exact same associative memory task and a similar acute physical exercise session^24^. The resulting estimated sample size was 15 participants, which corresponds to the exact sample size of a recent study using a similar exercising and biomarker protocol focusing on motor sequence memory^63^. In the present work, the data from 18 participants was used in the analyses. We agree that this may seem borderline for fMRI studies, but please note that we used a powerful within-subjects cross-over randomized design to maximize the statistical power from our sample similar to Marin Bosch^63^. Second, the fact that all included participants were regularly exercising healthy young male participants, may have induced a selection bias and may limit the generalizability of our findings. In the present study, we voluntarily chose to include only male participants because we wanted to test for not only moderate but also high intensity exercising within the same experimental design. This is mainly because 75% of maximal aerobic power output required for the high intensity condition does not correspond to the same exertion level across the hormonal cycle of women^64,65^, and for women taking contraceptive pills^64^. Therefore, including women would have led to approximations on the effort level, which would in turn have required that we considerably increased the sample size^66^. Additionally, blood lactate concentrations at our high intensity conditions also vary across the hormonal cycle^65^, and lactate changes have been shown to be linked to BDNF concentrations^67^ that we measure here. Lastly, regarding glucocorticoid response after physiological stress such as that associated with high intensity exercise, it significantly differs for men and women and, in women, depends on the phase of the hormonal cyclesee for example^68^ and on whether women take oral contraceptives^69^. These effects may influence fMRI responses acquired immediately after high intensity exercise, as we did in the present study^70^. Given the number and spacing of scanning sessions (about 2 weeks apart), it was unfortunately not possible to ensure that the moderate and intense exercising session were carried out at the same moment of the hormonal cycle of all our participants.

Further, we selected regularly exercising young volunteers to control for fatigue effects and ensure that all participants would complete the exercising protocols without exhaustion, but please note that a previous study showed similar effects of moderate intensity exercise using a more diverse population^24^. Although, high-intensity exercise could be performed by sedentary subjects, if exercise intensity is tailored individually to their fitness level, prolonged exercise at this intensity may cause notable discomfort, physiological stress and increased cortisol release^71,72^, which may overall hinder memory consolidation^48,49^. By applying strict inclusion criteria, we obtained a homogeneous sample of participants, but acknowledge that this may reduce the generalizability of our findings to the general population. Lastly, here we investigated AEA and BDNF levels in relation to associative memory, because these biomarkers are reportedly involved in hippocampal plasticity mechanisms and modulated by exercise. However, although we observed promising links between changes in biomarker levels, brain activations and associative memory performance, there are likely many other molecules involved in the effect of exercise on associative memory.

## Supplementary Information

Supplementary Information 1.

## Data Availability

The data that supports these findings and custom MATLAB codes used in this study are available from the corresponding author upon reasonable request.

## References

[1] Roig M, Nordbrandt S, Geertsen SS, Nielsen JB (2013). The effects of cardiovascular exercise on human memory: A review with meta-analysis. Neurosci. Biobehav. Rev..

[2] Lautenschlager NT (2008). Effect of physical activity on cognitive function in older adults at risk for Alzheimer disease: A randomized trial. JAMA.

[3] Wu CW (2007). Treadmill exercise counteracts the suppressive effects of peripheral lipopolysaccharide on hippocampal neurogenesis and learning and memory. J. Neurochem..

[4] Erickson KI (2011). Exercise training increases size of hippocampus and improves memory. Proc. Natl. Acad. Sci. USA.

[5] Boldrini M (2018). Human hippocampal neurogenesis persists throughout aging. Cell Stem Cell.

[6] Sorrells SF (2018). Human hippocampal neurogenesis drops sharply in children to undetectable levels in adults. Nature.

[7] Bekinschtein P, Oomen CA, Saksida LM, Bussey TJ (2011). Effects of environmental enrichment and voluntary exercise on neurogenesis, learning and memory, and pattern separation: BDNF as a critical variable?. Semin. Cell Dev. Biol..

[8] Vaynman S, Ying Z, Gomez-Pinilla F (2004). Hippocampal BDNF mediates the efficacy of exercise on synaptic plasticity and cognition. Eur. J. Neurosci..

[9] Rojas Vega S (2006). Acute BDNF and cortisol response to low intensity exercise and following ramp incremental exercise to exhaustion in humans. Brain Res..

[10] Neeper SA, Gomez-Pinilla F, Choi J, Cotman C (1995). Exercise and brain neurotrophins. Nature.

[11] Farmer J (2004). Effects of voluntary exercise on synaptic plasticity and gene expression in the dentate gyrus of adult male Sprague–Dawley rats in vivo. Neuroscience.

[12] van Praag H, Christie BR, Sejnowski TJ, Gage FH (1999). Running enhances neurogenesis, learning, and long-term potentiation in mice. Proc. Natl. Acad. Sci. USA.

[13] Dietrich A, McDaniel WF (2004). Endocannabinoids and exercise. Br. J. Sports Med..

[14] Sparling PB, Giuffrida A, Piomelli D, Rosskopf L, Dietrich A (2003). Exercise activates the endocannabinoid system. NeuroReport.

[15] Raichlen DA, Foster AD, Seillier A, Giuffrida A, Gerdeman GL (2013). Exercise-induced endocannabinoid signaling is modulated by intensity. Eur. J. Appl. Physiol..

[16] Hill MN (2010). Endogenous cannabinoid signaling is required for voluntary exercise-induced enhancement of progenitor cell proliferation in the hippocampus. Hippocampus.

[17] Chevaleyre V, Takahashi KA, Castillo PE (2006). Endocannabinoid-mediated synaptic plasticity in the CNS. Annu. Rev. Neurosci..

[18] Katona I, Freund TF (2012). Multiple functions of endocannabinoid signaling in the brain. Annu. Rev. Neurosci..

[19] Gomez-Gonzalo M (2015). Endocannabinoids induce lateral long-term potentiation of transmitter release by stimulation of gliotransmission. Cereb. Cortex.

[20] Talani G (2016). Enhanced glutamatergic synaptic plasticity in the hippocampal CA1 field of food-restricted rats: Involvement of CB1 receptors. Neuropsychopharmacology.

[21] Ferreira-Vieira TH, Bastos CP, Pereira GS, Moreira FA, Massensini AR (2014). A role for the endocannabinoid system in exercise-induced spatial memory enhancement in mice. Hippocampus.

[22] van Dongen EV, Kersten IH, Wagner IC, Morris RG, Fernandez G (2016). Physical exercise performed four hours after learning improves memory retention and increases hippocampal pattern similarity during retrieval. Curr. Biol..

[23] Tomporowski PD (2003). Effects of acute bouts of exercise on cognition. Acta Psychol. (Amst.).

[24] Marin Bosch B, Bringard A, Ferretti G, Schwartz S, Igloi K (2017). Effect of cerebral vasomotion during physical exercise on associative memory, a near-infrared spectroscopy study. Neurophotonics.

[25] Suwabe K (2018). Rapid stimulation of human dentate gyrus function with acute mild exercise. Proc. Natl. Acad. Sci..

[26] Szuhany KL, Bugatti M, Otto MW (2015). A meta-analytic review of the effects of exercise on brain-derived neurotrophic factor. J. Psychiatr. Res..

[27] Suwabe K (2017). Acute moderate exercise improves mnemonic discrimination in young adults. Hippocampus.

[28] Tomporowski PD, Ellis NR, Stephens R (1987). The immediate effects of strenuous exercise on free-recall memory. Ergonomics.

[29] Igloi K, Gaggioni G, Sterpenich V, Schwartz S (2015). A nap to recap or how reward regulates hippocampal-prefrontal memory networks during daytime sleep in humans. Elife.

[30] Labar G, Michaux C (2007). Fatty acid amide hydrolase: From characterization to therapeutics. Chem. Biodivers..

[31] Shah YB, Prior MJ, Dixon AL, Morris PG, Marsden CA (2004). Detection of cannabinoid agonist evoked increase in BOLD contrast in rats using functional magnetic resonance imaging. Neuropharmacology.

[32] Bekinschtein P (2013). BDNF in the dentate gyrus is required for consolidation of "pattern-separated" memories. Cell Rep.

[33] Yanagisawa H (2010). Acute moderate exercise elicits increased dorsolateral prefrontal activation and improves cognitive performance with Stroop test. Neuroimage.

[34] Glover GH, Li TQ, Ress D (2000). Image-based method for retrospective correction of physiological motion effects in fMRI: RETROICOR. Magn. Reson. Med..

[35] Birn RM, Smith MA, Jones TB, Bandettini PA (2008). The respiration response function: The temporal dynamics of fMRI signal fluctuations related to changes in respiration. Neuroimage.

[36] Dinges DF (1997). Cumulative sleepiness, mood disturbance, and psychomotor vigilance performance decrements during a week of sleep restricted to 4–5 hours per night. Sleep.

[37] Ellenbogen JM, Hu PT, Payne JD, Titone D, Walker MP (2007). Human relational memory requires time and sleep. Proc. Natl. Acad. Sci. USA.

[38] Liang JC, Preston AR (2017). Medial temporal lobe reinstatement of content-specific details predicts source memory. Cortex. J. Devot. Stud. Nervous Syst. Behav..

[39] Chadwick MJ (2016). Semantic representations in the temporal pole predict false memories. Proc. Natl. Acad. Sci. USA.

[40] Fuss J (2015). A runner's high depends on cannabinoid receptors in mice. Proc. Natl. Acad. Sci. USA.

[41] Hillard CJ (2018). Circulating endocannabinoids: From whence do they come and where are they going?. Neuropsychopharmacology.

[42] Molteni R, Ying Z, Gomez-Pinilla F (2002). Differential effects of acute and chronic exercise on plasticity-related genes in the rat hippocampus revealed by microarray. Eur. J. Neurosci..

[43] Bliss TV, Lomo T (1973). Long-lasting potentiation of synaptic transmission in the dentate area of the anaesthetized rabbit following stimulation of the perforant path. J. Physiol..

[44] Panja D, Bramham CR (2014). BDNF mechanisms in late LTP formation: A synthesis and breakdown. Neuropharmacology.

[45] Chang YK, Labban JD, Gapin JI, Etnier JL (2012). The effects of acute exercise on cognitive performance: A meta-analysis. Brain Res..

[46] Svedahl K, MacIntosh BR (2003). Anaerobic threshold: The concept and methods of measurement. Can. J. Appl. Physiol..

[47] ten Brinke LF (2015). Aerobic exercise increases hippocampal volume in older women with probable mild cognitive impairment: A 6-month randomised controlled trial. Br. J. Sports Med..

[48] Hill EE (2008). Exercise and circulating cortisol levels: The intensity threshold effect. J. Endocrinol. Invest..

[49] Het S, Ramlow G, Wolf OT (2005). A meta-analytic review of the effects of acute cortisol administration on human memory. Psychoneuroendocrinology.

[50] Mamounas LA (2000). BDNF promotes the regenerative sprouting, but not survival, of injured serotonergic axons in the adult rat brain. J. Neurosci..

[51] Moreira FA, Aguiar DC, Guimaraes FS (2007). Anxiolytic-like effect of cannabinoids injected into the rat dorsolateral periaqueductal gray. Neuropharmacology.

[52] Mei F, Nagappan G, Ke Y, Sacktor TC, Lu B (2011). BDNF facilitates L-LTP maintenance in the absence of protein synthesis through PKMzeta. PLoS One.

[53] Rex CS (2007). Brain-derived neurotrophic factor promotes long-term potentiation-related cytoskeletal changes in adult hippocampus. J. Neurosci..

[54] Bekinschtein P (2007). Persistence of long-term memory storage requires a late protein synthesis- and BDNF- dependent phase in the hippocampus. Neuron.

[55] Liu HL, Zhao G, Cai K, Zhao HH, Shi LD (2011). Treadmill exercise prevents decline in spatial learning and memory in APP/PS1 transgenic mice through improvement of hippocampal long-term potentiation. Behav. Brain Res..

[56] Levine ES, Crozier RA, Black IB, Plummer MR (1998). Brain-derived neurotrophic factor modulates hippocampal synaptic transmission by increasing N-methyl-D-aspartic acid receptor activity. Proc. Natl. Acad. Sci. USA.

[57] Rondi-Reig L (2006). Impaired sequential egocentric and allocentric memories in forebrain-specific-NMDA receptor knock-out mice during a new task dissociating strategies of navigation. J. Neurosci..

[58] Tsien JZ, Huerta PT, Tonegawa S (1996). The essential role of hippocampal CA1 NMDA receptor-dependent synaptic plasticity in spatial memory. Cell.

[59] Jackson MB (2013). Recall of spatial patterns stored in a hippocampal slice by long-term potentiation. J. Neurophysiol..

[60] Duzel E, van Praag H, Sendtner M (2016). Can physical exercise in old age improve memory and hippocampal function?. Brain J. Neurol..

[61] Basso JC, Shang A, Elman M, Karmouta R, Suzuki WA (2015). Acute exercise improves prefrontal cortex but not hippocampal function in healthy adults. J. Int. Neuropsychol. Soc..

[62] Birn RM, Diamond JB, Smith MA, Bandettini PA (2006). Separating respiratory-variation-related fluctuations from neuronal-activity-related fluctuations in fMRI. Neuroimage.

[63] Marin Bosch B (2020). Effect of acute physical exercise on motor sequence memory. Sci. Rep..

[64] Barba-Moreno L, Cupeiro R, Romero-Parra N, Jansede Jonge XAK, Peinado AB (2019). Cardiorespiratory responses to endurance exercise over the menstrual cycle and with oral contraceptive use. J. Strength Cond. Res..

[65] Mattu AT, Iannetta D, MacInnis MJ, Doyle-Baker PK, Murias JM (2020). Menstrual and oral contraceptive cycle phases do not affect submaximal and maximal exercise responses. Scand. J. Med. Sci. Sports.

[66] Clayton JA (2018). Applying the new SABV (sex as a biological variable) policy to research and clinical care. Physiol. Behav..

[67] Boyne P (2019). Exercise intensity affects acute neurotrophic and neurophysiological responses poststroke. J. Appl. Physiol..

[68] Merz CJ (2017). Contribution of stress and sex hormones to memory encoding. Psychoneuroendocrinology.

[69] Nielsen SE, Segal SK, Worden IV, Yim IS, Cahill L (2013). Hormonal contraception use alters stress responses and emotional memory. Biol. Psychol..

[70] Merz CJ (2012). Neuronal correlates of extinction learning are modulated by sex hormones. Soc. Cogn. Affect. Neurosci..

[71] Budde H, Machado S, Ribeiro P, Wegner M (2015). The cortisol response to exercise in young adults. Front. Behav. Neurosci..

[72] Tsai CL (2014). Executive function and endocrinological responses to acute resistance exercise. Front. Behav. Neurosci..

