# Supplementary material for: A single session of moderate intensity exercise influences memory, endocannabinoids and brain derived neurotrophic factor levels in men
Source: Sci Rep. 2021 Jul 13;11:14371. doi: 10.1038/s41598-021-93813-5 (PMC8277796; doi:10.1038/s41598-021-93813-5)
Supplement: Supplementary file 1 — Supplementary Information 1. [file 41598_2021_93813_MOESM1_ESM.docx]

# SUPPLEMENTARY INFORMATION

### METHODS

Participants: As literature shows that our biomarkers of interest may be affected by female hormonal cycles and by premenstrual syndrome[^1^](#_ENREF_1) and in order to homogenize exercising intensities, we recruited only male participants. All twenty participants were within the normal ranges on self-assessed questionnaires for depression (BDI[^2^](#_ENREF_2)), anxiety (STAI[^3^](#_ENREF_3)) circadian typology (PSQI[^4^](#_ENREF_4)). The rationale behind this selection procedure was that i) sedentary subjects may not be able to complete our high intensity condition and ii) the ventilatory threshold in highly trained individuals is known to be shifted towards 80-90% of maximal cardiac frequency (Fcmax). By including participants with a VO2max between 40 and 65, we thus ensured that our high intensity condition could be carried out by all participants and that it corresponded to an Exercising condition above the ventilatory threshold for all participants.

VO2max: A maximal incremental test was performed during an introductory visit, during which participants also performed a habituation session of the associative task. VO2max was measured using an electrically braked cycle ergometer (Ergometrics er800S, Ergoline, Jaeger, Germany). Respiratory gas flows and ventilation were continuously measured at the mouth on a breath-by-breath basis, using a metabolic unit (K4b^2^, Cosmed, Italy), consisting of a Zirconium Oxygen analyzer, an infrared CO2 meter and a turbine flowmeter. As recommended by the manufacturer, the gas analyzers were calibrated with ambient air and with a mixture of known gases (O_2_ 16 %, CO_2_ 5 %, N_2_ as balance), and the turbine by means of a 3-l syringe. Beat-by-beat heart rate (HR) was continuously monitored by cardiotachography (Polar RS 800 CX, Polar, Finland). Gas exchange variables (V˙O2, V˙CO2, V˙E, and RER) were continuously recorded on a breath-by-breath basis and later averaged over 10s sliding intervals for further analysis. The initial power output was 50W for 4min, followed by increases of 25W each 2min until 80% of maximal HR predicted by age was reached, then 25W increments each 1min until volitional exhaustion. The criteria for V˙O2max were RER > 1.1, plateau in V˙O2 (change of <100ml/min in the last three consecutive 20-s averages), and a HR within 10 beats/min of the maximal level predicted by age. Results of this test (see **Fig. S4** for VO2max distribution) were used to select power output for subsequent constant tests, based on the relationship between V’O2 and power output. Those participants with a VO2max between 40ml/kg/min and 65ml/kg/min were invited to come back for 3 experimental visits separated by one to two weeks, according to a within-subjects design with the three Exercising conditions (rest, moderate intensity exercise, high intensity exercise) randomly counterbalanced across participants. We report that 23 participants performed the VO2max, 2 of which did not meet the inclusion criteria.

Experimental visits: For each visit, participants were asked to keep a regular exercising schedule during at least 5 days before coming to the lab. Moreover, they were requested to refrain from intense physical activity for the 48h preceding the experimental visits. Compliance was documented by fitness tracker (Fitbit Charge HR, Fitbit, San Francisco, USA). Participants arrived at 08:00 AM on an empty stomach, and had a controlled breakfast with an experimenter consisting of coffee or tea, orange or apple juice, bread, and jam. Participants were allowed to eat as much as they desired but we controlled that they ate approximately similar amounts for all visits, they were allowed one caffeinated drink only. We did not allow them to eat any lipids to minimize inter-subject variability in endocannabinoid measures which heavily depend on lipid consumption.

Exercise conditions: For the two exercise conditions, participants pedaled on a cycle ergometer (Ergoline GmbH, Bitz, Germany), the pedaling frequency was kept between 60 and 80 cycles per minute, which was shown on a small screen in front of the participant. For both exercise conditions, the experimenters checked cardiac frequency every 3-5 minutes to adjust the resistance of the ergometer if necessary.

Rest condition: To minimize interference with memory, we carefully selected these magazines so that they were mainly composed of pictures, and that there was little to be learned from their content. We purposefully did not let participants watch a movie during rest to minimize motor imagery.

Associative memory task in fMRI: We adapted an associative memory task[^5^](#_ENREF_5)^,^[^6^](#_ENREF_6) consisting of two parts: encoding and test, separated by an exercise (moderate or high intensity) or rest period (**Fig. 1A**). To avoid interference across experimental visits for this within-subjects design, we showed different pictures belonging to three specific themes at each visit: “office”, “shoe shop” or “house” (one theme per visit). The pictures in each theme for the experimental visits were matched in difficulty and counterbalanced across Exercising conditions and visits (**Fig. 1B**). All the pictures were downloaded from Flickr Creative Commons without restrictions to reuse or modify and can be published under CC-BY open-access license. Note that for the habituation session of the task, participants had to memorize 5 series of a “swimming pool” theme.

During the encoding session, participants were first shown 8 series of 6 pictures one picture at a time (2000ms per picture), and were asked to encode each series as a whole (**Fig. 1B**). Then, they were trained on the 8 series 3 times, i.e. during three successive learning blocks. For each series, participants were shown the first picture of the series alone (e.g., pen, for the “office” theme; **Fig. 1B**) presented during 2000ms. Then, the same first picture was presented in the upper half of the screen together with two options for the second picture in the series (chair) in the lower half of the screen, one being the correct next picture and the other picture being from a different series (as depicted on the left panel of **Fig. 1C**). Participants could not answer for 2000ms, then the sentence “choose the next element” appeared on the screen and participants were instructed to press a button to give their answer when they had made their decision. Participants had to select the correct next picture by pressing a left or right button. The correct picture was then shown (providing a feedback for each trial), followed by this same picture together with the two next options for the third picture in the series (desk). This continued until the last picture in the series (office building). Additionally, two control series occurred pseudo-randomly during each block during which participants were shown a picture of a given color (red, green or blue) and had then to choose the picture of the same color (**Fig. 1D**). During each learning block, all 8 associative memory series and 2 control series were shown once. Stimuli were delivered and responses recorded using a MATLAB Toolbox (Cogent 2000, http://www.vislab.ucl.ac.uk/cogent_2000.php).

During the test session, participants were presented with one cue picture and two other pictures, among which they had to select the one belonging to the same series as the cue picture. The two options could represent the immediate next item in the series (direct trials) or could be separated by one or two items from the cue picture (inference of order 1 or order 2 trials; **Fig. 1C**). All types of trials were shown in a randomized order, and were presented in the same format and with the same timeframe as during learning, except that no feedback was provided. In this session, 16 trials of the control “color” task were also included (**Fig. 1D)**.

For the delayed retest session, all 18 participants came back for a surprise retest in fMRI 3 months after the last experimental visit. Participants did not know at test that there would be a retest session. The task was identical to the test sessions, except that pictures of all three themes were now mixed in a random order. For time constraints, we included for each of the eight sequences of pictures of all three themes (24 sequences) two direct trials, two inference order 1 trials and one inference order 2 trial (5 trials) totaling 120 trials overall.

Blood samples: Overall 12 ml of blood were collected before and after the rest or exercise periods. Seven ml of blood were collected into a BD Vacutainer clot-activator tube (CAT), allowed to clot for 30 minutes at room temperature and centrifuged at 1100g for 15 minutes at 4°C. Serum was collected from the supernatant in aliquots of 200μl and frozen at -80°C until analysis. The other 5ml of blood were collected into a BD Vacutainer K_2_EDTA 5.4mg tube and centrifuged immediately at 8009g for 10 min. Plasma was collected from the supernatant in aliquots of 200μl frozen at -80°C until analysis. All samples were centrifuged in a Heraeus Biofuge Stratos (ThermoFisher) centrifuge. The Quantikine ELISA Human Free BDNF kits (R&D sytems) were used to quantify serum BDNF via an enzyme-linked immunosorbent assay (ELISA) following the manufacturer’s instructions.

AEA and 2-AG were extracted from 100μl of plasma by liquid-liquid extraction, and then separated by liquid chromatography (Ultimate 3000RS, Dionex, CA, USA). Analyses were performed on a 5500 QTrap® triple quadrupole/linear ion trap (QqQLIT) mass spectrometer equipped with a TurboIon-SprayTM interface (AB Sciex, Concord, ON, Canada) as described previously[^7^](#_ENREF_7)^,^[^8^](#_ENREF_8).

Functional MRI data acquisition: T2*-weighted fMRI 2D images were obtained with a multiband gradient echo-planar sequence acquiring 3 slices at a time using axial slice orientation (66 slices; voxel size, 2 x 2 x 2mm; repetition time (TR) = 1880ms; echo time (TE) = 34ms; flip angle (FA) = 60°). A whole-brain structural image was acquired at the end of the first test part with a T1-weighted 3D sequence (192 contiguous sagittal slices; voxel size, 1.0 x 1.0 x 1.0mm; TR = 1900ms; TE = 2.27ms; FA = 9°). Continuous measures of heart rate and breathing rhythm were acquired using a Biopac (Biopac Systems, CA93117, USA).

Conventional fMRI analysis: This analysis included standard preprocessing procedures: realignment, slice timing to correct for differences in slice acquisition time, normalization (images were normalized to an MNI template), and smoothing (with an isotropic 8-mm FWHM Gaussian kernel) – except for the decoding analysis where we used unsmoothed images (see below). While scanning was not performed right after physical exercise or rest, but about 1h later, we nevertheless performed corrections to regress out potential physiological artifacts from heart rate and breathing using Retroicor^[9](#_ENREF_9)^ and RVHcorr[^10^](#_ENREF_10)^,^[^11^](#_ENREF_11), respectively.

For contrasts of interest, we then applied small volume correction (SVC) using a hippocampal mask [as in Grande et al.^12^](#_ENREF_12) created within the Anatomy toolbox of SPM12 [SPM Anatomy toolbox 2.2, Forschungszentrum Jülich GmbH^13^](#_ENREF_13) and retained activated clusters with a minimal size of 10 contiguous voxels within the SVC mask. Coordinates of brain regions are reported in MNI space.

Decoding analysis: A decoding procedure was performed on unsmoothed data. For each session of each participant, we extracted the timeseries of all voxels within the bilateral hippocampus region of interest, which was defined in the Anatomy toolbox as the union of CA1, CA2, CA3 and DG regions. Timeseries were detrended and demeaned, then the movement parameters obtained from realignment and breathing parameters from Retroicor and RVHcorr were regressed out. For each visit of each participant, the peak of the BOLD response (corresponding to 6 seconds after the onset of the trial) was extracted for each single trial from each voxel of the hippocampal ROI to obtain a ‘voxel by single trial matrix'. Then, we averaged these voxel-wise values per trial type (hits, errors, control) and Exercising condition (rest, mod, high), thus yielding an average activity pattern for each trial type (i.e., voxel by trial-type matrix) and Exercising condition. We applied a decoding procedure to test how well trial types (hits, misses, control) could be predicted from these hippocampal voxel-based activation patterns for each Exercising condition separately, [see van Dongen^14^](#_ENREF_14) for a similar approach. Decoding accuracy was estimated by applying a leave-one-out procedure, including a standard cross-validation procedure for each trial of the left out participant. Thus, for each trial-type and each Exercising condition, we obtained the proportion of trials that the decoding procedure has classified correctly or incorrectly (as hits, misses, or control). From these measures we derived estimates of sensitivity (true positive rate or, for each trial-type, the proportion of trials correctly classified as belonging to this trial-type) and specificity (true negative rate or, for each trial-type, the proportion of trials correctly classified as not belonging to this trial-type). Please note that we found no effect for specificity estimates, and therefore focused on sensitivity estimates in the results and discussion sections. To assess whether there was a laterality effect in the hippocampus, we subsequently ran analyses using the left and right hippocampus as separate regions of interest (see **Fig. S3**).

### SUPPLEMENTARY FIGURES


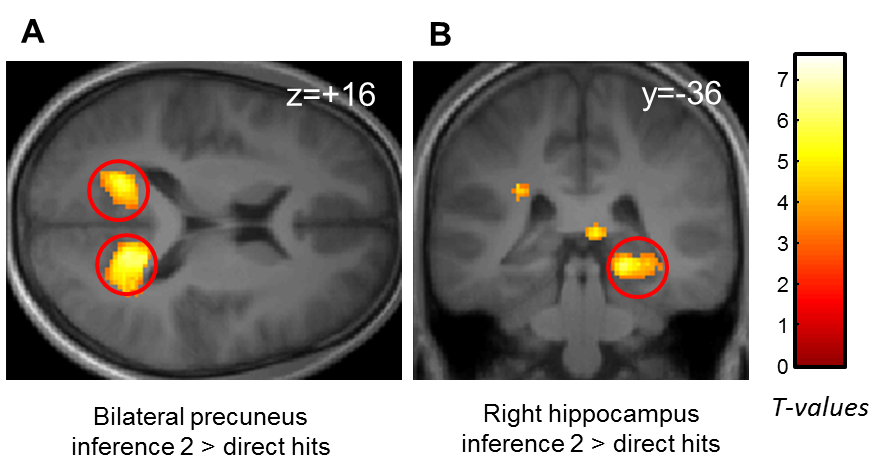


### Fig. S1. Brain correlates of increasing Relational distance

A) Bilateral precuneus activation for increasing Relational distance (inference 2 hits > direct hits). B) Right hippocampal activation for increasing Relational distance (inference 2 hits > direct hits) [z score=4.35 (18, -38, -8), p<0.05 SVC].


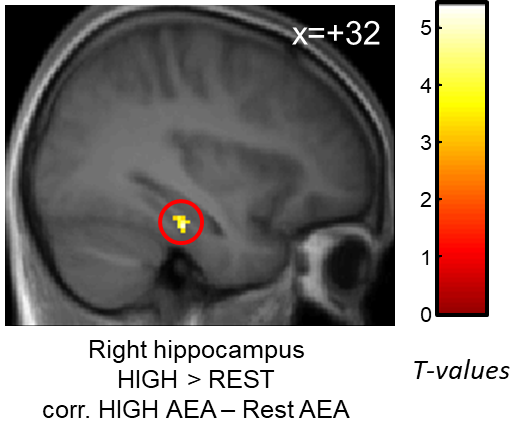


### Fig. S2. Hippocampal response correlated with endocannabinoid increase after high intensity exercise

Increased right parahippocampal (extending into hippocampus) response [z-score=4.08 (32, -24, -18), p<0.05 SVC] for hits after high intensity exercise compared to hits after rest correlated with the increase in AEA level after high intensity exercise.

Activation map displayed on the mean T1 anatomical scan of the whole population.


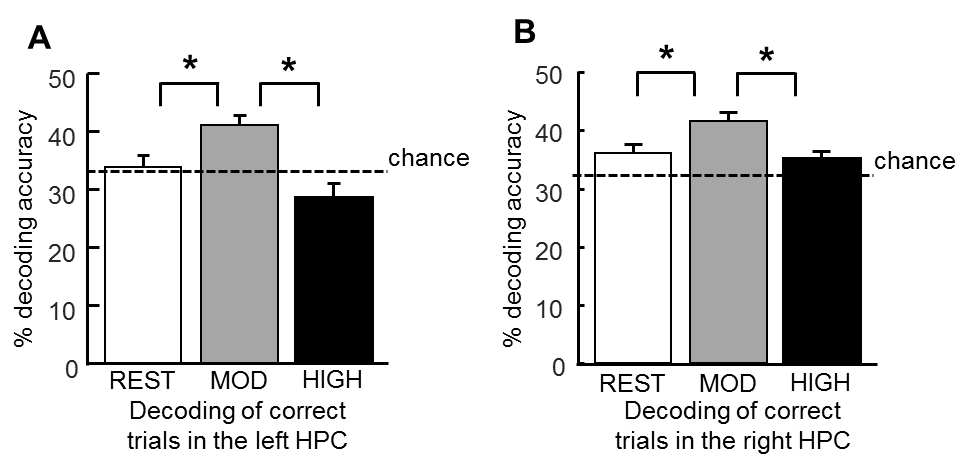


### Fig. S3. Better decoding accuracy in both left and right hippocampi after moderate intensity exercise

**A)** Better sensitivity of decoding of correct trials in the left hippocampus after moderate exercise than rest and high intensity exercise. ANOVA F(2, 34)=7.60, p=0.002, post-hoc p_mod-rest_=0.03 p_mod-high_=0.001. Decoding after both rest and high intensity exercise is not different from chance level (both p>0.05), while being above chance level after moderate intensity exercise (t(17)=4.38, p<0.001). B) Better sensitivity of decoding of correct trials in the right hippocampus after moderate exercise than rest and high intensity exercise. ANOVA F(2, 34)=5.24, p=0.01, post-hoc p_mod-rest_=0.01 p_mod-high_=0.01. Decoding after both rest and high intensity exercise is not different from chance level (both p>0.05), while it is above chance level after moderate intensity exercise (t(17)=5.15, p<0.001).


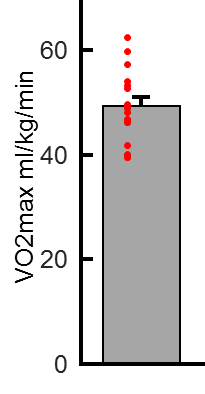


### Fig. S4. Distribution of VO2max measures


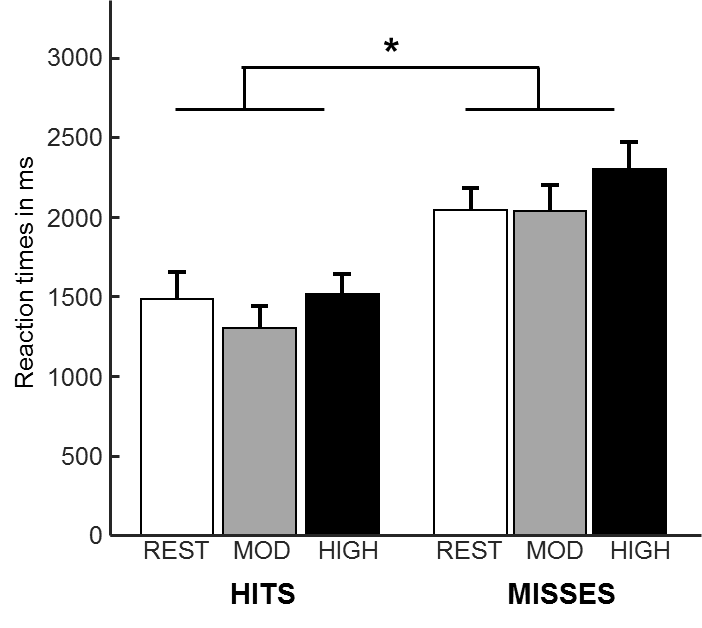


### Fig. S5. Reaction times

Reaction times as a function of response type and Exercising condition.

### REFERENCES

1 Cubeddu, A. *et al.* Brain-derived neurotrophic factor plasma variation during the different phases of the menstrual cycle in women with premenstrual syndrome. *Psychoneuroendocrinology* **36**, 523-530, doi:10.1016/j.psyneuen.2010.08.006 (2011).

2 Beck, A. T., Epstein, N., Brown, G. & Steer, R. A. An inventory for measuring clinical anxiety: psychometric properties. *Journal of consulting and clinical psychology* **56**, 893-897 (1988).

3 Spielberger, C. D., Gorsuch, R. L., Lushene, R., Vagg, P. R. & Jacobs, G. A. *Manual for the State-Trait Anxiety Inventory*. (1983).

4 Horne, J. A. & Ostberg, O. A self-assessment questionnaire to determine morningness-eveningness in human circadian rhythms. *International journal of chronobiology* **4**, 97-110 (1976).

5 Igloi, K., Gaggioni, G., Sterpenich, V. & Schwartz, S. A nap to recap or how reward regulates hippocampal-prefrontal memory networks during daytime sleep in humans. *Elife* **4**, doi:10.7554/eLife.07903 (2015).

6 Marin Bosch, B., Bringard, A., Ferretti, G., Schwartz, S. & Igloi, K. Effect of cerebral vasomotion during physical exercise on associative memory, a near-infrared spectroscopy study. *Neurophotonics* **4**, 041404, doi:10.1117/1.NPh.4.4.041404 (2017).

7 Quercioli, A. *et al.* Improvement in coronary circulatory function in morbidly obese individuals after gastric bypass-induced weight loss: relation to alterations in endocannabinoids and adipocytokines. *Eur Heart J* **34**, 2063-2073, doi:10.1093/eurheartj/eht085 (2013).

8 Thomas, A., Hopfgartner, G., Giroud, C. & Staub, C. Quantitative and qualitative profiling of endocannabinoids in human plasma using a triple quadrupole linear ion trap mass spectrometer with liquid chromatography. *Rapid Commun Mass Spectrom* **23**, 629-638, doi:10.1002/rcm.3918 (2009).

9 Glover, G. H., Li, T. Q. & Ress, D. Image-based method for retrospective correction of physiological motion effects in fMRI: RETROICOR. *Magnetic resonance in medicine* **44**, 162-167 (2000).

10 Birn, R. M., Diamond, J. B., Smith, M. A. & Bandettini, P. A. Separating respiratory-variation-related fluctuations from neuronal-activity-related fluctuations in fMRI. *Neuroimage* **31**, 1536-1548, doi:10.1016/j.neuroimage.2006.02.048 (2006).

11 Birn, R. M., Smith, M. A., Jones, T. B. & Bandettini, P. A. The respiration response function: the temporal dynamics of fMRI signal fluctuations related to changes in respiration. *Neuroimage* **40**, 644-654, doi:10.1016/j.neuroimage.2007.11.059 (2008).

12 Grande, X. *et al.* Holistic recollection via pattern completion involves hippocampal subfield CA3. *J Neurosci*, doi:10.1523/JNEUROSCI.0722-19.2019 (2019).

13 Eickhoff, S. B. *et al.* A new SPM toolbox for combining probabilistic cytoarchitectonic maps and functional imaging data. *Neuroimage* **25**, 1325-1335, doi:10.1016/j.neuroimage.2004.12.034 (2005).

14 van Dongen, E. V., Kersten, I. H., Wagner, I. C., Morris, R. G. & Fernandez, G. Physical Exercise Performed Four Hours after Learning Improves Memory Retention and Increases Hippocampal Pattern Similarity during Retrieval. *Curr Biol* **26**, 1722-1727, doi:10.1016/j.cub.2016.04.071 (2016).
